# From Inner Topological Structure to Functional Nanofibers: Theoretical Analysis and Experimental Verification

**DOI:** 10.3390/membranes11110870

**Published:** 2021-11-12

**Authors:** Dan Tian, Chunhui He

**Affiliations:** 1 School of Science, Xi’an University of Architecture and Technology, Xi’an 710055, China; 2 School of Civil Engineering, Xi’an University of Architecture and Technology, Xi’an 710055, China; mathew_he@yahoo.com

**Keywords:** nanofiber, topological structure, Hall–Petch effect, nanoparticle, bubble-electrospinning

## Abstract

The mechanical strength of spider silk is the highest among all natural fibers, and its flexibility is also excellent; this phenomenon can be explained geometrically, due to its hierarchical structure, the last cascade of which beginning with well-ordered macromolecules. The inner topological structure of a nanofiber plays an important role in controlling its functions, e.g., its mechanical, electrical and chemical properties. This paper shows that nanoparticles can be well-ordered in the electrospinning process as a result, the nanofibers’ properties can be adjusted. Some experiments are designed to verify our theoretical prediction.

## 1. Introduction

“The structure of the universe is the most perfect and the wise creation of God”; as pointed out by Leonhard Euler (1707–1783), nature is full of geometrical inherence. There is increasing evidence that the inner topological structure can endow various remarkable functions of a material. At the same time, some microstructures of animals and plants in nature lead us to obtain more and more discoveries and developments in scientific research. The most significant feature of such structures is the decomposition of a large and complex system into several unidirectionally dependent levels, relying, then, on each level, itself, and the synergy between them to bring unexpected advantages to materials [1,2,3,4,5]. Among natural fibers, silk, spun by silkworms and spiders, is a typical representative of natural soft materials with a hierarchical structure [6,7,8]. Spider silk has a typical micro-level hierarchy structure; each strand of spider silk contains a large number of nanofibers, which means that spider silk is a combination of countless nanofibers, and these nanofibers exhibit various physical and biological properties that affect the performance of spider silk. It is considered to be one of the most promising materials due to their excellent mechanical properties, like biocompatibility, thermal conductivity, and controllable solubility [9,10]. In addition, spider-silk materials are used in many engineering fields [11,12]. However, due to the difficulty in obtaining spider silk, researchers have tried various methods of preparing materials that mimic spider silk. In addition to using biological methods to extract spider-silk proteins to prepare biomimetic materials, many researchers have also put substantial effort into preparing materials with hierarchical structures based on the natural hierarchical structure of spider silks. Kurnsteiner et al. [13] locally controlled nanoprecipitation and martensitic transformation during the manufacturing process, resulting in the formation of a multi-scale microstructure hierarchy; their material had a tensile strength of 1300 MPa and elongation capacity of 10%. Bonderer et al. [14], by using the principles of natural composites, showed that by assembling sub-micron-thick ceramic sheets hierarchically in a tough polymer matrix, a layered hybrid film with high composite properties can be obtained. Aizenberg et al. [15] arranged nanometer-scaled silica spheres in well-defined microscopic concentric rings, glued together by an organic matrix, to form laminated spicules; this structure showed outstanding mechanical rigidity and stability. These studies show that when a material is modified in terms of structure, especially when it has a hierarchical structure, some of the properties of the material will also change accordingly, and this change may be very beneficial to the application of the material. Therefore, in order to improve the mechanical properties of nanofibers, we have aimed to prepare nanofibers with a hierarchical structure.

Inspired by the structure of spider silk and our previous research on the internal structure of nanofibers, in this work, based on the Hall–Petch effect and bubble-electrospinning, we controlled the internal structure of the bubble wall similarly to spider silk, thereby improving the properties of the obtained nanofibers. We also studied the influence of nanoparticles on bubble morphology and nanofibers through the addition of carbon nanoparticles to the polymer solution, exploring the relationship between the inner topological structure and the functional performance of the nanofibers through theoretical analysis.

## 2. Hall–Petch Effect and Wool’s Inner Topological Structure

The Hall–Petch effect is often used in materials science to adjust the hardness, durability or ductility of materials [16,17,18]. Hall–Petch effect is written as,

(1) $$\sigma =\sigma_{0}+\frac{k}{d^{\beta }}$$

where σ is the elastic modulus or strength, $\sigma_{0}$ is the bulk property, *k* is a material constant, *d* is the mean grain size, and β is a scaling parameter.

**Figure 1:**
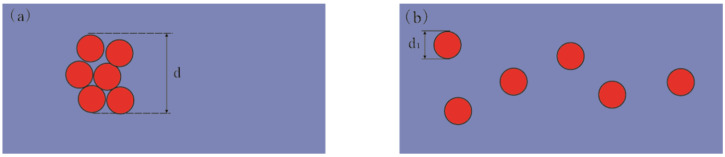
Hall–Petch effect for (**a**) coagulated nanoparticles and (**b**) well-ordered nanoparticles.

As shown in Figure 1, the particles agglomerated and dispersed in the prepared fiber. In the experiment, when particles coagulate together, the diameter of the particle is equal to the diameter of the agglomerate (because the coagulated particle is then regarded as a new large particle); but when the particles are dispersed and distributed, the diameter of each particle is its own diameter. Here, the particle diameter used in the calculation is the average of all particle diameters. According to the Hall–Petch effect, that is, the formula given above, we can see that the smaller the diameter (*d*) of the particle, the larger the σ, and the mechanical properties of the material will be greatly improved. Therefore, we considered that if we could disperse the nanoparticles during the experiment, the mechanical properties of the nanofibers should also be greatly improved.

**Figure 2:**
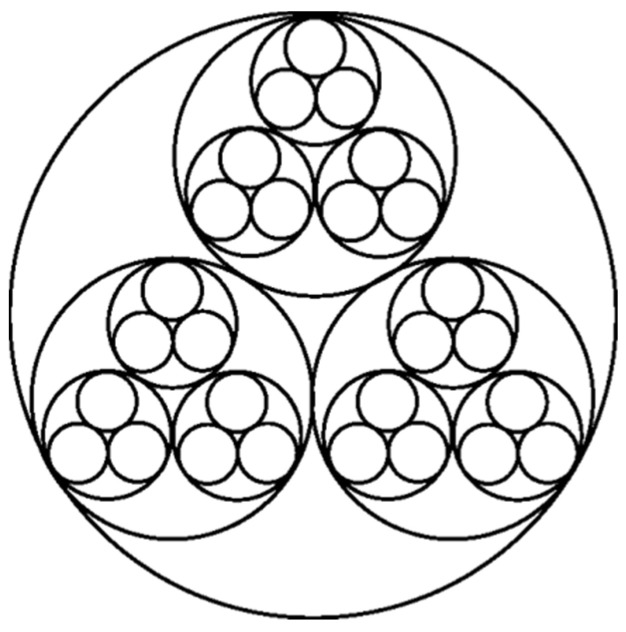
Microstructure of wool.

On the other hand, as for topological structure, fiber wool has a typical topological fractal structure and its fractal dimension is close to 1.618, as shown in Figure 2. This structure of wool gives it excellent thermal properties and excellent unidirectional thermal properties [19]. Thus, if we can control the arrangement of nanoparticles or macromolecules by bubble electrospinning to have a fractal topology similar to wool, the various properties of the prepared nanofibers will also share these changes.

## 3. Geometric Potential Theory for Inner Topological Structure

When fluid molecules move chaotically or randomly, it leads to a turbulent flow, and a single molecule’s energy is dissipated by its adjacent molecules. If all moving molecules are well-ordered, like those in a tornado, great energy can be produced. According to the geometric potential theory, every molecule or nanoparticle can produce a short-range force; when all molecules or nanoparticles are in a good order, all the short-range forces at the small scale can produce a large force at the large scale, just like ferromagnetic ordering (see its explanation in [20]). The chaotic distribution of crystals results in poor mechanical properties, while an ordered distribution leads to good mechanical properties.

## 4. Experimental Design

**Figure 3:**
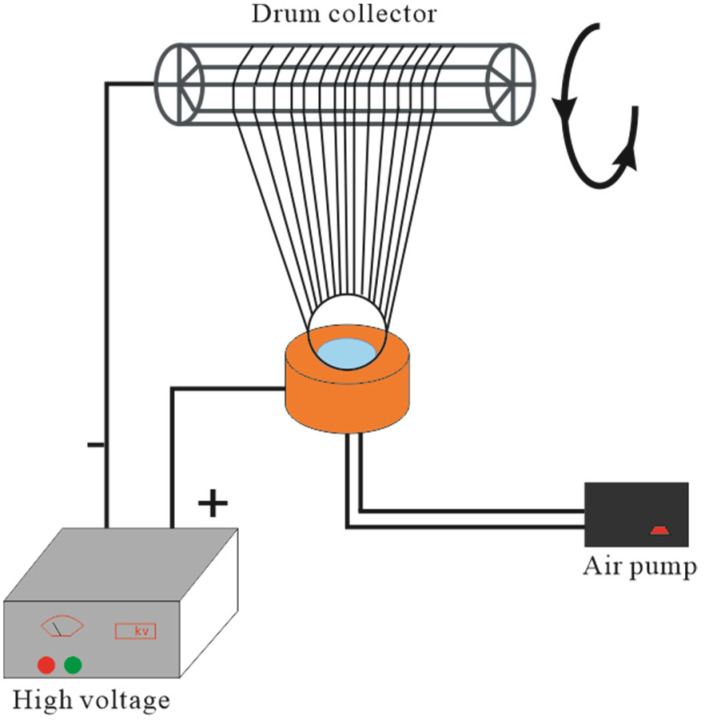
Bubble elctrospinning device.

Bubble electrospinning [21,22,23] was used in our experiment; the experiment setup is illustrated in Figure 3. The nozzle diameter was 4 cm, the voltage was 40 kV, the temperature was 20 °C, the relative humidity was 48%, and the distance between the nozzle and receptor was 26 cm.

## 5. Experimental Verification

### 5.1. Materials and Instruments

Polyvinyl alcohol (PVA) was purchased from Shanghai Aladdin Biochemical Technology Co., Ltd. (Shanghai, China), and used as received. The alcoholysis degree of the PVA was 97.5–99.0%, and it was stored at room temperature.

Carbon nanoparticles were purchased from Shanghai Aladdin Biochemical Technology Co., Ltd. (Shanghai, China), and used as received; the average diameter of nanoparticles was 100 nm.

### 5.2. Instrumentation

Electrospun nanofibrous morphologies were analyzed using a S4800 Cold Field Scanning electron microscope (SEM, Hitachi S-4800, Tokyo, Japan). Fibers for SEM analysis were collected on aluminum foil, mounted on an SEM disc, and sputter-coated with an 8-nm Pt/Au layer to reduce electron charging effects. The nanofibrous diameter distributions were analyzed with ImageJ software (National Institute of Mental Health, Bethesda, MD, USA).

The mechanical properties studies were performed with an INSTRON-3365 material testing machine (INSTRON Company, Norwood, MA, USA). The thickness of the fibers’ membrane were measured by micrometer; each sample was measured three times, and their mean values were calculated.

### 5.3. Solution Preparation

Fibres were placed in 4.8 g of PVA in a beaker, then dissolved in 55.2 g deionized water. The beaker was sealed and put it onto the heating magnetic stirrer (DF-101S, Xinrui Instrument Inc, Beijing, China) for stirring; the temperature of the water was 80 °C. After the PVA was completely dissolved into a homogeneous solution, it was blended into a solution with 8 wt% PVA concentration. Then we added carbon nanoparticles to the PVA solution and stirred the solution with a high-speed stirrer. The qualities of the nano-carbon powders were 0.5 g, 1 g, 1.5 g, 2 g, respectively.

## 6. Results and Discussion

### 6.1. Bubble Morphology

**Figure 4:**
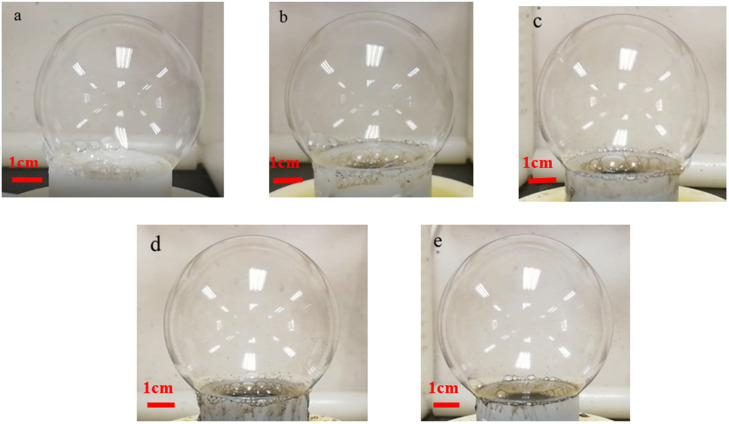
Bubble produced in bubble-electrospinng (Spinning solutions with different amounts of carbon nanoparticles). (**a**) 0 g, (**b**) 0.5 g, (**c**) 1 g, (**d**) 1.5 g, (**e**) 2 g.

**Figure 5:**
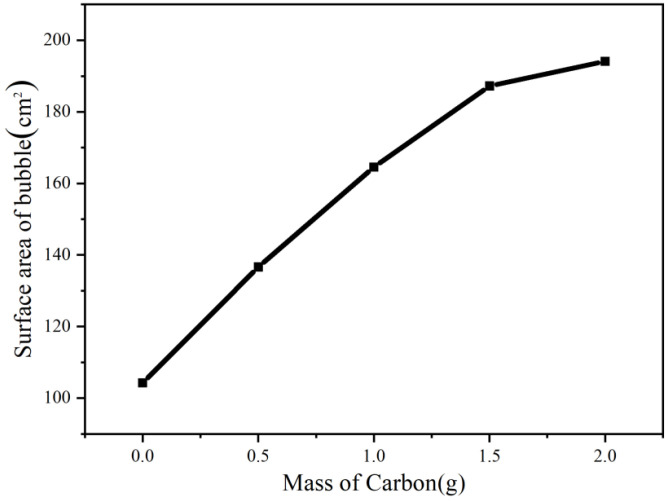
The changing trend of bubble size.

**Figure 6:**
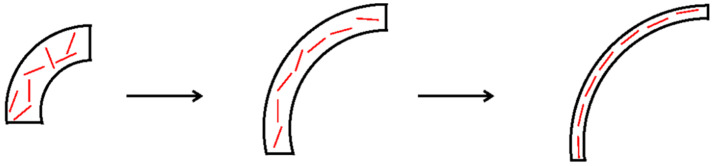
The arrangement of nanoparticles in the bubble wall.

In the process of bubble electrospinning, we first studied the change of bubble size after adding different amounts of nanoparticles without electricity. As shown in Figure 4 and Figure 5, we can see that, as the amount of nanoparticles increased, the bubbles became larger. Obviously, with the same amount of liquid, the larger the bubble, the thinner the bubble wall. According to our previous research [18] and the Hall–Petch effect, when the thickness of the bubble wall tends toward hundreds of nanometers, the nano-effect arises [24], and, according to the nano-effect and the VdW attraction between the alkyl polymer chains and the carbon, the bubble wall should become very strong at this time, such that the bubbles would not burst easily. On the other hand, Figure 6 shows that, as the bubble became larger and the bubble wall became thinner, the internal nanoparticle or arrangements of macromolecular chains changed. It can be seen from the figure that when more nanoparticles were added, the bubble walls became stronger, the bubbles became more stable, and larger bubbles could be obtained. At the same time, the arrangement of macromolecular chains in the polymer became more and more orderly. According to the Hall–Petch effect, this orderly arrangement of nanoparticles and macromolecular chains should increase the strength of the bubble wall. Under this circulation, the time for which bubbles remain stable should increase, which is very beneficial in bubble electrospinning, because, in bubble electrospinning, jets generated when bubbles are stable will continuously produce nanofibers. The longer bubbles remain stable, the higher the spinning efficiency. On the other hand, the more orderly the arrangement of the nanoparticles in the bubble, the more orderly arrangement of the particles in the prepared nanofibers, which should have a certain impact on the mechanical properties of the obtained nanofibers.

### 6.2. Morphological Characterization (SEM) of Nanofiber

**Figure 7:**
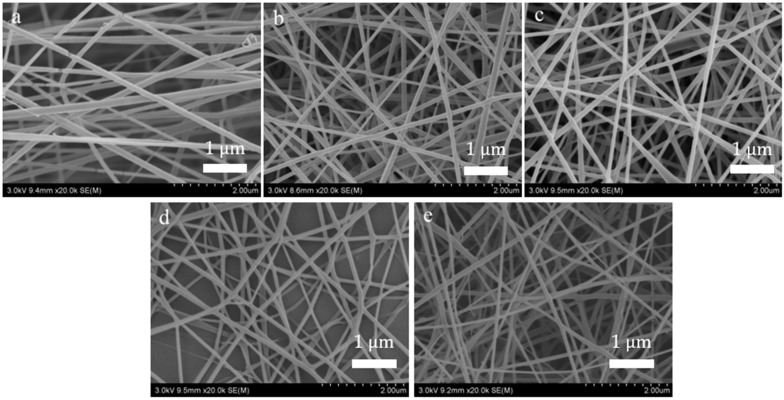
SEM of nanofiber, (**a**) PVA, (**b**) PVA with 0.5 g carbon nanoparticles, (**c**) PVA with 1 g carbon nanoparticles, (**d**) PVA with 1.5 g carbon nanoparticles, (**e**) PVA with 2 g carbon nanoparticles.

**Figure 8:**
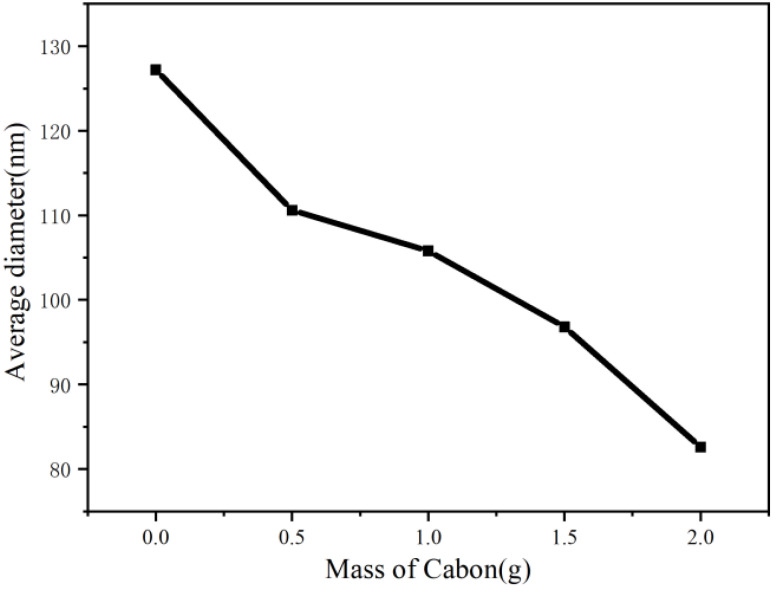
The changing trend of nanofiber’s diameter.

Figure 7 and Figure 8 show the morphology and average diameter changes of different nanofibers prepared by bubble spinning, respectively. From the above bubble morphology, it is easy to observe that, with the increased quantity of nanoparticles, the bubbles in the bubble-electrospinning process became increasingly larger, whereupon the jet generated by the bubble surface became finer, leading to thinner final nanofibers. On the other hand, when the bubble became larger, the bubble wall became thinner, so the nanofibers obtained from the stretched fragments after the bubble burst would inevitably be thinner. The combination of these reasons suggests that with the increase in the amount of added nanoparticles, the prepared nanofibers would be thinner. The surface of all nanofibers were smooth.

### 6.3. Machine Property Test of Nanofiber Membranes

**Figure 9:**
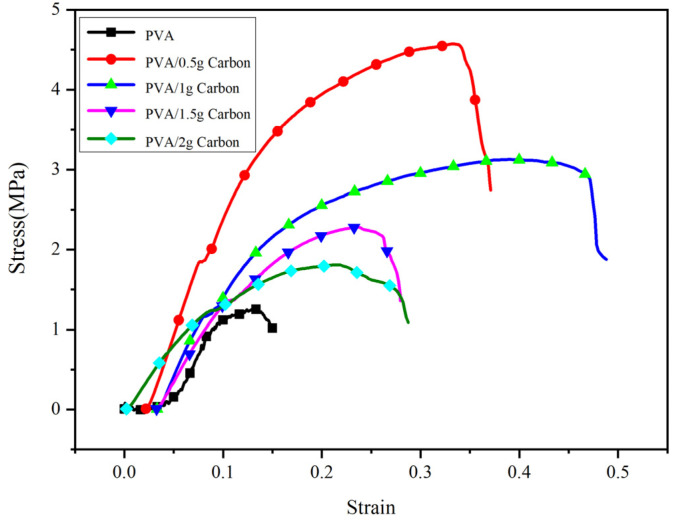
Mechanical property of the nanofiber membrane.

Figure 9 shows the mechanical properties tests of different nanofiber membranes prepared by bubble electrospinning. With increasing amounts of added nanoparticles the mechanical properties of the nanofiber membranes exhibited a pattern of change. For 0.5 g of added nanoparticles, the mechanical properties of the nanofiber membranes were the greatest. This is because after adding the nanoparticles, the mechanical properties of the nanofiber membranes increased according to the Hall–Petch effect. We can also see from the figure that the mechanical properties of the nanofiber membranes with nanoparticles were always better than pure-PVA nanofiber membranes. For those nanofiber membranes with nanoparticles, as the amounts of nanoparticles increased, the mechanical properties of the nanofiber membranes showed a decreasing trend in quality. This is because more nanoparticles create larger bubbles in the bubble spinning process, and, for enlarged bubbles, the nanoparticles in the bubble wall were orderly arranged. However, because the bubbles were very large, for the same area of bubble wall, larger bubbles contain fewer nanoparticles than smaller bubbles. According to the Hall–Petch effect, the mechanical properties of the nanofiber membranes worsen, consequently.

## 7. Conclusions

In this paper, in order to imitate the internal structure of spider silk, we used nanoparticles to adjust the size of the bubbles in an bubble-electrospinning process, to further control the orderly arrangement of nanoparticles in the bubble wall and thereby improve the bubble-wall strength, increase the duration of bubbles’ stability, and to adjust the properties of the prepared nanofiber membranes. Our experiments and theoretical analysis showed that when more nanoparticles were added, bubbles became larger, the nanoparticles in bubbles’ walls were orderly arranged, the strength of bubbles’ walls increased, bubbles remained stable longer and the bubble-electrospun yield was higher. Additionally, the mechanical properties of the nanofiber membranes were improved after the addition of nanoparticles, such that smaller amounts of nanoparticles produced better mechanical properties in the nanofiber membranes.
